# The Historic Built Environment As a Long-Term Geochemical Archive: Telling the Time on the Urban “Pollution Clock”

**DOI:** 10.1021/acs.est.3c00153

**Published:** 2023-07-12

**Authors:** Katrin Wilhelm, Jack Longman, Christopher D. Standish, Tim De Kock

**Affiliations:** † Oxford Resilient Buildings and Landscapes Laboratory (OxRBL), School of Geography and the Environment, University of Oxford, South Parks Road, Oxford, OX1 3QY, U.K.; ‡ Marine Isotope Geochemistry, Institute for Chemistry and Biology of the Marine Environment (ICBM), University of Oldenburg, Carl-von-Ossietzky-Str. 9-11, 26129 Oldenburg, Germany; § Department of Geography and Environmental Sciences, Northumbria University, Newcastle-upon-Tyne, NE1 8ST, United Kingdom; ∥ School of Ocean & Earth Sciences, University of Southampton, National Oceanography Centre, European Way, Southampton, SO14 3ZH, U.K.; ⊥ Antwerp Cultural Heritage Sciences (ARCHES), Faculty of Design, University of Antwerp Blindestraat 9, 2000 Antwerp, Belgium

**Keywords:** urban pollution, Pb isotope ratios, coal burning, black crusts, paleopollution, heavy metals, limestone, laser ablation ICP-MS

## Abstract

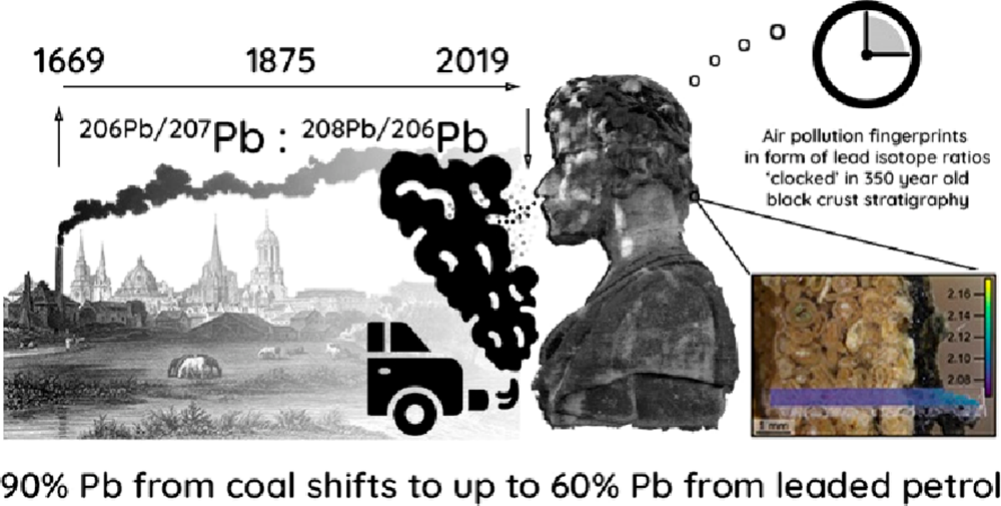

This study introduces a novel methodology for utilizing historic built environments as reliable long-term geochemical archives, addressing a gap in the reconstruction of past anthropogenic pollution levels in urban settings. For the first time, we employ high-resolution laser ablation mass spectrometry for lead isotope (^206^Pb/^207^Pb and ^208^Pb/^206^Pb) analysis on 350-year-old black crust stratigraphies found on historic built structures, providing insights into past air pollution signatures. Our findings reveal a gradual shift in the crust stratigraphy toward lower ^206^Pb/^207^Pb and higher ^208^Pb/^206^Pb isotope ratios from the older to the younger layers, indicating changes in lead sources over time. Mass balance analysis of the isotope data shows black crust layers formed since 1669 primarily contain over 90% Pb from coal burning, while other lead sources from a set of modern pollution including but not limited to leaded gasoline (introduced after 1920) become dominant (up to 60%) from 1875 onward. In contrast to global archives such as ice cores that provide integrated signals of long-distance pollution, our study contributes to a deeper understanding of localized pollution levels, specifically in urban settings. Our approach complements multiple sources of evidence, enhancing our understanding of air pollution dynamics and trends, and the impact of human activities on urban environments.

## Abstract 2

This paper contributes a novel approach to developing a more reliable systematic methodology for utilizing black crusts as long-term geochemical archives with a finer-scale resolution for urban pollution.

## Introduction

The geochemical content of natural archives such as ice, marine sediment, and peat are often studied to investigate changing levels of anthropogenic pollution through time.^1−3^ Such studies exploit, among other tracers, the immobility of lead (Pb) in the environment,^4,5^ interpreting changing Pb concentrations and/or isotopic ratios as indicators of changing levels and/or sources of air pollution.^6,7^ An additional archive that is less frequently examined is stone weathering crusts. Pb concentrations in weathering crusts of historic urban built structures typically range from ten to hundreds of parts per million (ppm), reaching up to thousands of ppm when in the vicinity of industrial sites.^8−10^ This poses problems when Pb, together with other carcinogenic and toxic components, is remobilized and reintroduced into the overall urban pollution budget through processes such as cleaning (e.g., sandblasting), fire, weathering of surface coating such as lead-based paint, and common surface erosion.^10−13^ Indeed, recent studies on urban heavy metal dust have found an increasing risk to the environment and human health due to such remobilization of Pb.^14−17^ Therefore, there is an urgent need to advance our understanding of the interactions between the historic built environment and both ongoing and past environmental pollution accumulated in weathering crusts.

Both the definition of what constitutes a crust and its varied morphology descriptions raise some issues, as the field does not use a consistent terminology. In general, a weathering crust (which might also be referred to as a case hardening, damage layer, etc.) results from surface and subsurface biophysicochemical alterations of the host stone substrate and is common in polluted urban environments.

This study focuses on “black crusts” as a subgroup of weathering crusts. Black crusts are promoted through atmospheric sulfur dioxide (SO_2_) and acidic water and form on calcareous and Ca-rich substrates as superficial gypsum layers which are distinct from the host stone/rock and often incorporate particulate matter, polyaromatic hydrocarbons (PAH), and heavy metals.^18−24^ While it is recognized that gypsum alteration can also occur in the host substrate, these alterations are typically distinct and lack the deposition of airborne particles and their associated black discoloration. This study focuses on the deposition of the pollutants and their characteristic fingerprints in black crusts only (and not on mineral alterations).

Black crusts on historic urban built structures hold great potential as valuable geochemical archives for advancing the understanding of urban air pollution processes. Recent research has investigated their use as nonselective passive samplers for atmospheric pollution.^25−27,8,10,28,29^ However, despite the emergent recognition of their potential, there is currently no well-defined protocol or standardized method established for fully utilizing black crusts as reliable outdoor archives for urban air pollution.^10^ Furthermore, the field of study lacks cohesion due to a lack of integration of previous research. Additionally, current research often fails to capture the finer-scale resolution pollution record stored in the stratigraphy of the black crusts, which is crucial for reconstructing past air pollution conditions and comparing them to other archives of paleopollution like lake sediments or bogs (e.g., Mighall et al. 2006^30^). In light of these gaps, we propose a methodology for high-resolution Pb isotope analysis of black crusts by laser ablation multicollector inductively coupled mass spectrometry (LA-MC-ICP-MS). Our aim is to establish black crusts as a reliable outdoor record for monitoring the changing levels and patterns of urban air pollution. To demonstrate these methods, we present a case study focusing on black crusts that formed on historic sculptures in Oxford, UK.

Our objectives were: (1) to establish a finer-scale chronology in the crust stratigraphy using Pb isotope analysis in combination with ICP-MS for the first time, (2) to assess the potential of black crusts as accurate nonselective passive samplers for atmospheric pollution, and (3) to provide recommendations for optimal conditions to effectively utilize black crusts as geochemical archives. Finally, these findings are integrated in the wider context of black crust studies to both highlight key aspects that need to be considered and documented when retrieving and analyzing such samples and to firmly establish this geochemical archive as a useful long-term environmental record of urban air pollution. While our methodology focuses on limestone crusts, it holds potential applicability to various stone types.

These findings have broader implications for stone weathering research, conservation measures, and the promotion of healthy and sustainable cities. By establishing black crusts as reliable and informative records of urban air pollution, we can contribute to better understanding the long-term environmental impacts and developing effective strategies for pollution management and urban planning.

## Materials and Methods

### Sampling Strategy

**Figure 1:**
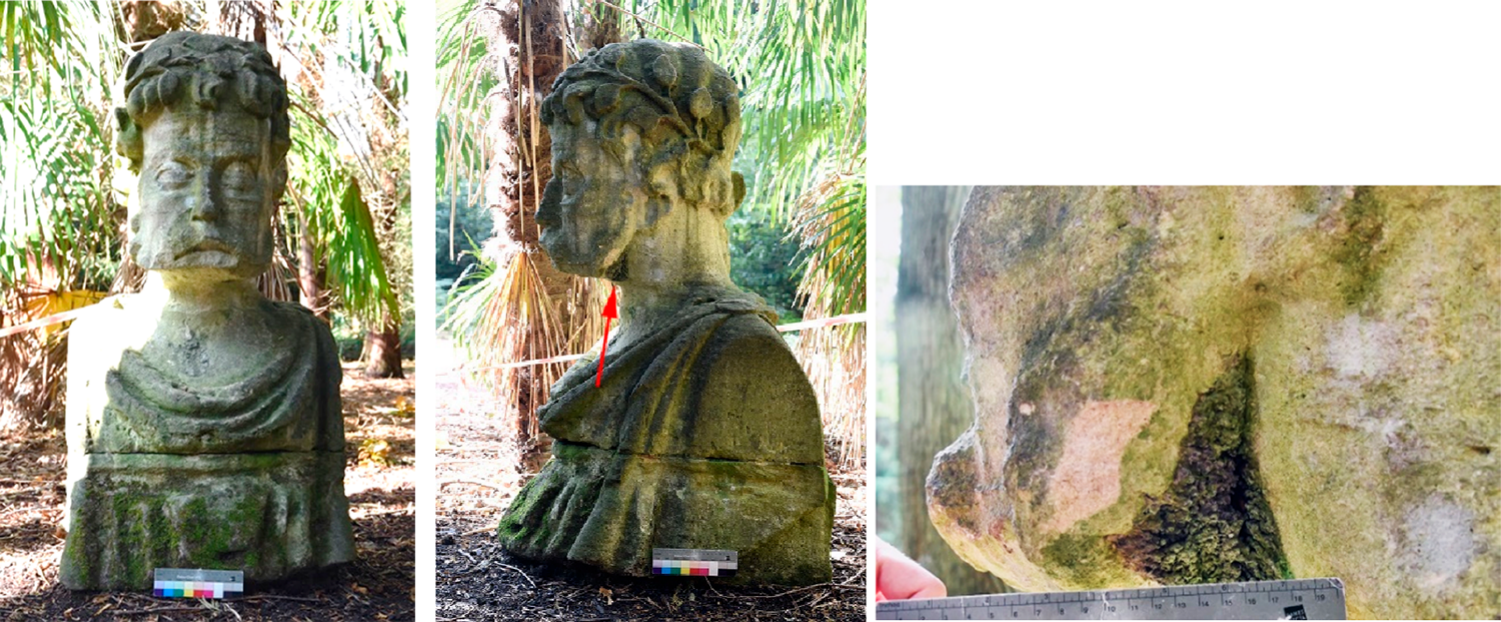
Left: Harcourt Arboretum (HAR), second generation stone head sculpture 2 (51° 41′0.1782″N 1° 11′58.0194″W). Currently faces SW. Middle and right: crust sampling region in sheltered area (red arrow indicates sampling area).

This study builds on a previous study which took advantage of a unique succession of three generations of stone head sculptures (Figures 1, S1, and S2) surrounding the Sheldonian Theatre on Broad Street in the city center of Oxford (51°45′15″N 1°15′18″W; altitude ∼64 m; annual average rainfall 681 mm;^31^ Köppen climate classification Cfb and Cfc^32^) which had been replaced twice previously, in 1886 (second generation) and 1972 (current generation), since the theater was built in 1668.^28^ Subsequent transportation of the sculptures to less polluted areas means that their “pollution clock” effectively stopped after periods exposed to air pollution. Therefore, these samples have been selected to investigate the potential for a benchmarking and identify distinct signatures (“fingerprints”) within the crust stratigraphy.^33^

This study analyzed a subset of samples suitable for LA-MC-ICP-MS analysis and included both the first generation heads in Malvern (**MAL**) and Worcester College Garden (**WOR**) as well as the second generation heads in Harcourt Arboretum (**HAR**). Table 1 shows a summary of the samples’ context (location, exposure period, etc.).

**Table 1:** Summary of Sample Subset Respective Locations, Current Aspect (the Historic Aspect of the Sample Location Has Been the Same), Limestone Type, Sculpture Generation, and Date of Installation^a^

Current location	Type of current location	Sample facing historic aspect	Sample facing current aspect	Generation	Date of installation on Broad St	Former location	Limestone type	Crust morphology	Date of sampling
Malvern (MAL)	Deep Rural	N	S.W.	1st	1669	SHE	Taynton	F/L	27.11.2019
Worcester College (WOR)	“Green” Urban	N	E	1st	1679–83	HSM	Taynton	F/L	27.01.2020
Harcourt Arboretum (HAR)	Rural urban	N	SE and N.E.	2nd	1875	HSM	Milton	F	25.09.2019

a SHE = Sheldonian Theatre, HSM = History of Science Museum, F = Framboidal crust morphology, L = Laminar crust morphology.

### Laser Ablation Multicollector Inductively Coupled Plasma Mass Spectrometry

Black crusts and sections of the host stone were analyzed for their Pb isotope ratios and Pb concentrations by LA-MC-ICP-MS, using a Neptune Plus MC-ICP mass spectrometer (Thermo Fisher Scientific, Waltham, MA, USA) equipped with 9 Faraday cup detectors and a central ion counter coupled to an Elemental Scientific Lasers (Bozeman, MT, USA) NWR193 excimer laser ablation system with a TwoVol2 ablation chamber. Samples were first sectioned using an HC sintered diamond rotating saw, then both samples and standards were mounted in MetPrep EpoFLO high-purity epoxy resin and polished to reveal a flat and smooth surface using Kemet PSU-M polishing cloths (grades 15 μm, 9 μm, 3 μm, 0.3 μm). Finally, the samples and standards were cleaned with alcohol in an ultrasonic bath.

Analytical protocols broadly followed those of Standish et al. (2013).^34^ Tune parameters were optimized for stability, sensitivity, and low oxide production (^254^(UO)+/^238^U+ < 1%) while ablating silicate reference material NIST SRM 610. Operating conditions are detailed in Table 2. NIST SRM612 and basaltic glass reference material BCR-2G were run as internal consistency standards. The laser was operated in transect mode, with a laser beam of 25 μm × 100 μm in area employed for samples and NIST glasses (where 25 μm is the dimension in the direction of travel). BCR-2G basaltic glass reference material was analyzed with a larger laser beam (100 × 150 μm) due to its lower Pb concentration. Typical sensitivities for NIST SRM610 were 5 V on ^208^Pb. Prior to data collection, samples were preablated to remove any surface contamination (repetition rate of 20 Hz, laser tracking speed of 200 μm/s, power density of 0.8 J/cm^–2^).

**Table 2:** Operating Conditions for LA-MC-ICP-MS

Instrument	
Mass Spectrometer	Thermo Scientific Neptune Plus multicollector inductively coupled plasma mass spectrometer
Laser Ablation System	Elemental Scientific Lasers NWR193 excimer laser ablation system with a TwoVol2 ablation chamber
RF Power	1400 W
Cones	Nickel X skimmer; jet sample

Cup Configuration						
	Cup	L2	C	H1	H2	H3
	Mass	^202^Hg	^204^Pb	^206^Pb	^207^Pb	^208^Pb
	Resistor	10^12^Ω	10^12^Ω	10^11^Ω	10^11^Ω	10^11^Ω
Integration time (s)	1.049					

Gas Flows	
Cooling Gas (Ar)	16 L min^–1^
Auxiliary Gas (Ar)	0.7 L min^–1^
Make-up gas (Ar)	1.0 L min^–1^
Ablation cell carrier gas (He)	0.7 L min^–1^
Additional Gas (N)	0.007 L min^–1^

Ablation Conditions	
Laser power density	∼6 J cm^–2^
Laser repetition rate	Samples and NIST glasses: 10 Hz BCR-2G: 20 Hz
Laser beam size	Samples and NIST glasses: 25 by 100 μm BCR-2G: 100 by 150 μm
Laser tracking speed	5 μm s^–1^
Ablation mode	Line

Standard data were collected over 200 integration cycles of 1.049 s (henceforth referred to as “data cycles” to emphasize the sequential acquisition of data points along the analyzed path or transect, revealing spatial variations in elemental concentrations and isotopic ratios within the sample); a period of 210 s. Sample data were also collected using 1.049 s integration cycles, but the number of data cycles was controlled by the thickness of the mineral crusts.

It is crucial to note that the crust thickness is only roughly correlated to the exposure time. Although a thicker crust may indicate a longer exposure time, it is the distinct layers with different isotopic fingerprints that offer a more accurate representation of the historical timeline. The isotopic composition of each layer reveals specific periods of pollution and environmental change regardless of the crust’s overall thickness. The presence of these successive layers with unique fingerprints is the key to understanding the chronology of air pollution rather than solely relying on the thickness of the crust.

An on-peak gas blank was analyzed immediately before and after ablation over 45 cycles of 1.049 s. All corrections were applied offline. Dynamic blank corrections were applied on all masses cycle by cycle, assuming a linear relationship between the preceding and succeeding blank measurements. Instrumental drift and mass bias were corrected by standard-sample bracketing to glass reference material NIST SRM610 and the values of Baker et al. (2004).^35^ For standard analyses, data cycles falling outside the 2SD of the mean were omitted. For sample analysis, data cycles were first screened for rare trips of the faraday detectors, and screening for outliers was not performed due to their heterogeneous nature.

To demonstrate internal precision, external reproducibility, and accuracy of the analytical setup, silicate reference material NIST SRM612 (∼39 ppm Pb) and basaltic glass reference material BCR-2G (∼11 ppm Pb) were run as secondary standards (ST 1). Internal precision on the ^206^Pb/^207^Pb and ^208^Pb/^206^Pb, expressed as two relative standard errors (S.E.) of the mean of the data cycles comprising one analysis, are <100 ppm for NIST SRM612, and typically <200 ppm for BCR-2G. Mean (±2SD) ^206^Pb/^207^Pb is 1.1026 ± 0.0002 (*n* = 4) for NIST SRM612 and 1.2000 ± 0.0005 (*n* = 4) for BCR-2G; mean (±2SD) ^208^Pb/^206^Pb is 2.1645 ± 0.0005 (*n* = 4) for NIST SRM612 and 2.0649 ± 0.0003 (*n* = 4) for BCR-2G. Mean (±2SD) Pb concentrations are 41.8 ± 3.8 ppm for NIST SRM612 and 11.8 ± 6.1 ppm for BCR-2G. These are comparable to published values.^35^

#### Solution MC-ICP-MS

The Pb isotope ratio compositions of two crusts (**HAR A S2 and MAL A S2**) were also characterized by solution MC-ICP-MS, as a further demonstration of accuracy for the laser ablation MC-ICP-MS approach. Crusts were sampled using a hand-held Dremel drill and 500 μm drill-bit. Powders (∼25 mg) were dissolved in concentrated HNO_3_–HCl (ratio 1:1), with samples refluxed overnight to ensure total digestion. Pb was isolated by ion exchange chromatography (using Biorad AG1-X8 resin) after conversion to bromide form with HBr.^36^ Pb isotope ratios were measured on a Neptune MC-ICP mass spectrometer, with samples corrected for instrumental mass fractionation using the ^207^Pb–^204^Pb SBL74 double spike.^37^ Samples were split into two aliquots immediately prior to measurement, one of which was spiked with SBL74 such that ^204^Pb_sample_/^204^Pb_spike_ was 0.1–0.2. The natural and double spiked fractions were then run in separate batches, at a target concentration of 20 ppb Pb. SRM NIST981 values achieved during the analytical sequence were ^206^Pb/^207^Pb = 1.093192 ± 22, and ^208^Pb/^206^Pb = 2.167068 ± 18, (uncertainties are 2SD in the last decimal place; *n* = 4).

Solution MC-ICP-MS gave the following results for the crust samples: ^206^Pb/^207^Pb = 1.117667 ± 2, ^208^Pb/^206^Pb = 2.143097 ± 2 for **HAR A S2**; and ^206^Pb/^207^Pb = 1.176077 ± 6, ^208^Pb/^206^Pb = 2.087121 ± 18 for **MAL A S2** (uncertainties are 2SE in the last decimal place). A single LA-MC-ICP-MS analysis was performed immediately adjacent to the location where each of the solution samples was drilled using the methods detailed above, the lengths of the laser ablation transects matching the diameter of the drill hole to best sample the same crust stratigraphies. Results were as follows: ^206^Pb/^207^Pb = 1.117524 ± 0.010423, ^208^Pb/^206^Pb = 2.144232 ± 0.009982 for **HAR A S2**; and ^206^Pb/^207^Pb = 1.177154 ± 0.004308, ^208^Pb/^206^Pb = 2.086947 ± 0.004295 for **MAL A S2** (uncertainties are 1SD). The two methods are therefore in agreement, further demonstrating the accuracy of the laser ablation approach.

#### Isotopic and Elemental Mapping

Isotopic and elemental mapping was performed in R^38^ using an adapted script from Chalk et al. (2021).^39^ Each laser line for ^206^Pb/^207^Pb, ^208^Pb/^206^Pb, and Pb concentration in ppm was first subjected to a 3SD rejection to remove any outliers and then a moving average was used to smooth the data. The width of the moving average window was 5 points for all data. The sets of five separate smoothed laser lines were mapped onto an equal spaced grid using their X and Y spatial coordinates from Elemental Scientific Lasers NWR193 excimer laser ablation system. The dimensions of the grid were governed by the resolution of the data and was constructed using the Raster package^40^ utilizing the “filledcontour” function. The X–Y resolution of the images produced was approximately 5 × 100 μm per pixel.

### Crust Archive Methodology Development

To aid the methodological development to establish black crusts as more reliable outdoor geochemical archives, we conducted an in-depth literature review of former studies addressing common challenges such as the datum point, mobility of trace elements within the crust, growth rate, and account of crust layers. As search engines for the literature review, we used Google Scholar, Web of Science, and Semantic Scholar; the latter is supported by artificial intelligence. From the wide range of literature, we screened 100 publications for studies either employing Pb isotope analysis or LA-ICP-MS on black crusts on limestone-built heritage (and similar terms such as “damage or weathering layer”, “gypsum-rich coatings”, “transformation layer”, “encrustation”, “exocrust”, rock coatings, accretions, patina^21,41−47^) as well as reporting on crucial factors such as crust morphology, aspect, height, orientation, conservation history, etc. (for a full list of relevant factors compare ST2).

## Results and Discussion

LA-MC-ICP-MS The mean Pb concentrations of the **first generation** crusts are 188.8 ± 7.3 ppm for **MAL A S1** (Table 2, 93.6 ± 4.5 ppm for **MAL A S2**, and 77.3 ± 5.7 ppm for **WOR A** (uncertainties are expressed as 2SE). The two crusts from Malvern are characterized by similar Pb isotope ratio compositions: **MAL A S1** gives a mean ^206^Pb/^207^Pb of 1.1771 ± 0.0001 and a mean ^208^Pb/^206^Pb of 2.0876 ± 0.0001, while **MAL A S2** gives a mean ^206^Pb/^207^Pb of 1.1779 ± 0.0002 and mean ^208^Pb/^206^Pb of 2.0871 ± 0.0002. **WOR A** is characterized by a mean ^206^Pb/^207^Pb of 1.1550 ± 0.0012 and mean ^208^Pb/^206^Pb of 2.1067 ± 0.0012. The **second generation** crusts are characterized by higher Pb concentrations with lower ^206^Pb/^207^Pb and higher ^208^Pb/^206^Pb: for **HAR A S2**, the mean Pb concentration is 604.9 ± 21.6 ppm, mean ^206^Pb/^207^Pb is 1.1077 ± 0.0002, and mean ^208^Pb/^206^Pb is 2.1539 ± 0.0002.

**Figure 2:**
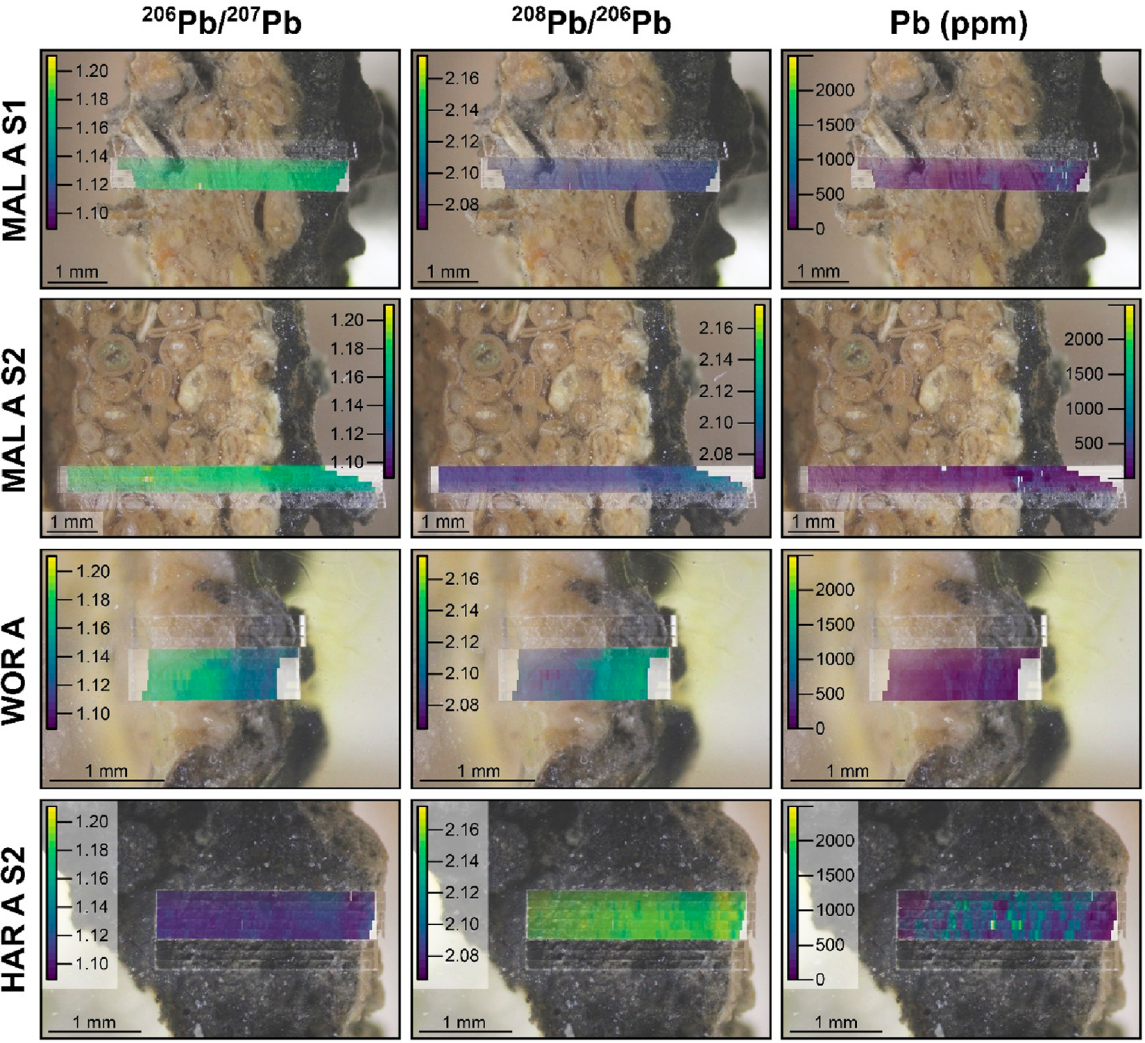
Cross sections of stone head sculpture crust samples from Malvern (MAL A S1 and MAL A S2), Worcester College (WOR A), and Harcourt Arboretum (HAR A S2), superimposed with Pb isotope and concentration maps. In all examples, the most recent/youngest crust layer is to the right side of the image.

The results suggest a shift in the source(s) of Pb exploited in Oxford between the time when the first and second generation crusts mineralized; those that formed on stonework installed in the 19th Century CE were exposed to a greater amount of Pb originating from a source characterized by lower ^206^Pb/^207^Pb and higher ^208^Pb/^206^Pb ratios, compared to those installed in the 17th Century CE. The Pb isotope ratio signature and Pb concentrations of the four crusts are presented in Figure 2 as mapped images.

**Figure 3:**
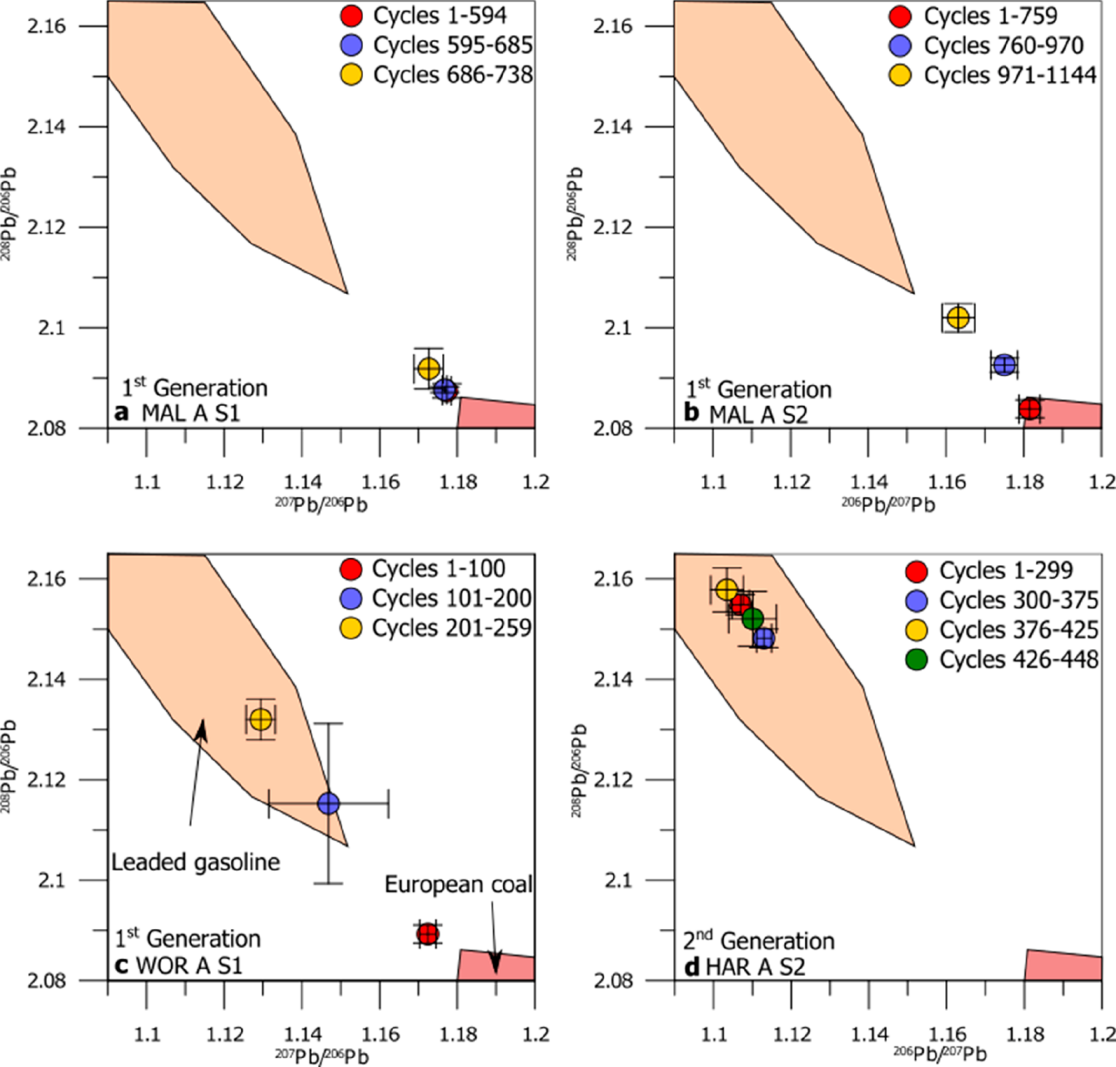
Graphs show the lead (^208^Pb/^206^Pb and ^206^Pb/^207^Pb) isotope ratios for four different samples from the 1st and 2nd generation stone head sculptures (WOR A S1 = Worcester College (first gen); HAR A S2= Harcourt Arboretum (2nd gen.); MAL A S1 and S2 = Malvern 1st gen.). The regions (“fingerprints”) indicating European coal and leaded gasoline are based on previous studies.^6,49^ Except for HAR A S2, which has a nonlinear history (forgotten some of the pollution clock time), the consecutive numbers of the data cycles correspond with the consecutive build up from older to younger crust. The “clock” in these examples is represented by isotopic fingerprints, which reveal specific pollution periods and environmental changes.

The high-resolution analysis performed here allows, for the first time, a detailed look at how the Pb source(s) evolved as each black crust mineralized. For example, for all first generation crusts, we find a gradual shift from the older to the younger layers of the crust stratigraphy toward lower ^206^Pb/^207^Pb and higher ^208^Pb/^206^Pb, indicating that black crusts record changes in lead sources over time (Figure 2). Each data cycle (’cycle’ in Figure 3) represents an individual layer within the crust; however, there is not a linear relationship between the layer thickness and the exposure period corresponding with a certain pollution fingerprint.

Previous research has shown that the layer thickness of black crust can vary significantly depending on the host stone substrate and its propensity to respond to a given environment. For example, for the same crust a range between 20 and 600 μm of growth rates have been reported.^41,48^ Rather than relying solely on a depth-to-time correlation, the timing of specific periods of pollution and environmental change in these examples is determined by the isotopic fingerprints present in each layer. These fingerprints offer valuable insights into the temporal occurrence of pollution events, enabling a more nuanced understanding of the dynamics and changes over time.

The samples **MAL A S1** and **MAL A S2** from the first generation head sculpture exposed since 1668 and now located in rural Malvern show for data cycles 1–594 (∼3.1 mm thickness) a ^206^Pb/^207^Pb ratio of **1.177 ± 0.001 (1SD)** and for data cycles 1–759 (∼4.0 mm thickness) a ^206^Pb/^207^Pb ratio of **1.182 ± 0.004 (1SD)**, respectively. This is similar to the isotopic composition of data cycles 760–970 (∼1.1 mm thickness) in **MAL A S2 1.175 ± 0.002 (1SD)** and data cycles 1–100 (∼0.5 mm thickness) in **WOR A S1 1.172 ± 0.002 (1SD)**. This isotopic composition is indicative of Pb released during European coal combustion as well as ore smelting (and other Pb containing materials, e.g., Galena) prior the 19th Century CE.^49,50^ This suggests these regions of the crust stratigraphy are representative of crustal growth between the emplacement of the heads (1669), and prior to their removal in 1869. These cycles reflect the oldest layers of the crusts nearest the host stone.

The Pb isotope ranges for ore smelting from southwest England overlap with coal signatures, as shown in Figure S3. This suggests that ore smelting may have contributed to the lead content in the crusts studied, although only to a limited extent, since Oxford did not have a direct smelting industry. Moreover, the nearest smelting regions are located at significant distances from Oxford, further reducing their influence. In the Middle Ages, lead was smelted in the Pennines and Mendips, with the latter being closer to Oxford at approximately 100 km in linear distance.^51^ While Zoltai et al. (1988^52^) found above-background concentrations of Pb up to 100 km away from smelting sources, other researchers reported distances of 40–65 km from the source.^50,53,54^ Meanwhile, coal usage as a fuel source began to expand more widely in England during the late 16th and early 17th centuries.^55−57^ Furthermore, in our previous publication, from which this study’s subset of samples is derived,^28^ our principal component analysis identified a simultaneous loading of Arsenic (As), Selenium (Se), and Titanium (Ti). These trace metals have been associated with coal burning in various studies.^25,58−62^

Data cycles 971–1144 (∼0.9 mm thickness) of **MAL A S2**, the outermost layers, show a ^206^Pb/^207^Pb ratio of **1.163 ± 0.003 (1SD)** which indicates a shift in Pb isotope ratios. This may represent the first shift of Pb source away from coal-dominance and further indicate a mix of Pb ratios where import of ore from Broken Hill, Australia introduces lower Pb ratios.^63,64^

The outer layers of **WOR A S1** (first gen.) show ^206^Pb^/207^Pb ratios of **1.147 ± 0.016 (1SD)** and **1.129 ± 0.004 (1SD)** for data cycles 101–220 (∼0.5 mm thickness) and 201–259 (∼0.3 mm thickness) respectively, consistent with the signature of leaded gasoline.^65^ This is an interesting observation as this first generation stone head had been removed in 1869 (long before the leaded gasoline was introduced in the 1930s.^49^ However, this suggests one of two possible exposure scenarios: either (1) the stone head may have been exposed to traffic pollution before it was placed in Worcester College Garden, or more likely (2) the garden’s proximity to busy roads (Walton Street < 100 m, Botley Road <130 m, and the nearby train station <450 m, linear distance) continue to be a source of pollution today.^66,67^ Pollution will be carried to the college garden and accumulate on the stone surface since it is close to major roads and downwind from the direction of the predominant wind, which is from S/SW to N/NE (meteoblue.com; cf., ref (68)). However, the surrounding walls (∼4–5m in height^69^) and trees (among the Worcester College Garden’s diverse tree community feature three “Champion trees” between 7.6–27 m tall) result in a reduction of the overall pollution amount.^70−72^ Therefore, this first generation stone head shows a “fingerprint” for leaded petrol, in contrast to the first generation stone heads in Malvern (a clean-air area), which do not.

The crust layers closest to the host stone in **HAR A S2**, the second generation crust (installed in 1875), show varying ^206^Pb^/207^Pb ratios from **1.107 ± 0.002 (1SD)** for data cycles 1–299 (∼1.6 mm thickness) and then **1.113 ± 0.001 (1SD)** for data cycles 300–375 (∼0.4 mm thickness), followed by **1.103 ± 0.004 (1SD)** for data cycles 376–425 (∼0.3 mm thickness) and finally **1.110 ± 0.004 (1SD)** for data cycles 426–448 (∼0.1 mm thickness). These are all consistent with a source dominated by gasoline.

The fact that this sample does not show a coal signature is explained through the assumption that we are dealing with a secondary crust that has formed on a surface that had lost its original surface (i.e., the oldest layers of the initial crust). The second generation of stone heads has been known for reacting to air pollution badly with signs of decay shortly after their installation (cf., ref (73), p 15), compared the appearance of the stone heads to “*illustrations in a medical textbook on skin diseases*”. Thus, it is highly likely that the crust portion that did contain the coal fingerprint had already been lost when a younger crust was formed. The absence of any ^206^Pb^/207^Pb ratios greater than **1.120** is consistent with the stone head’s weathering history, which was removed in the 1970s as leaded gasoline was being phased out in Europe since the 1980s^23^ and transported to the Harcourt Arboretum’s clean air environment where no further (modern) pollution accumulated.^49^

**Figure 4:**
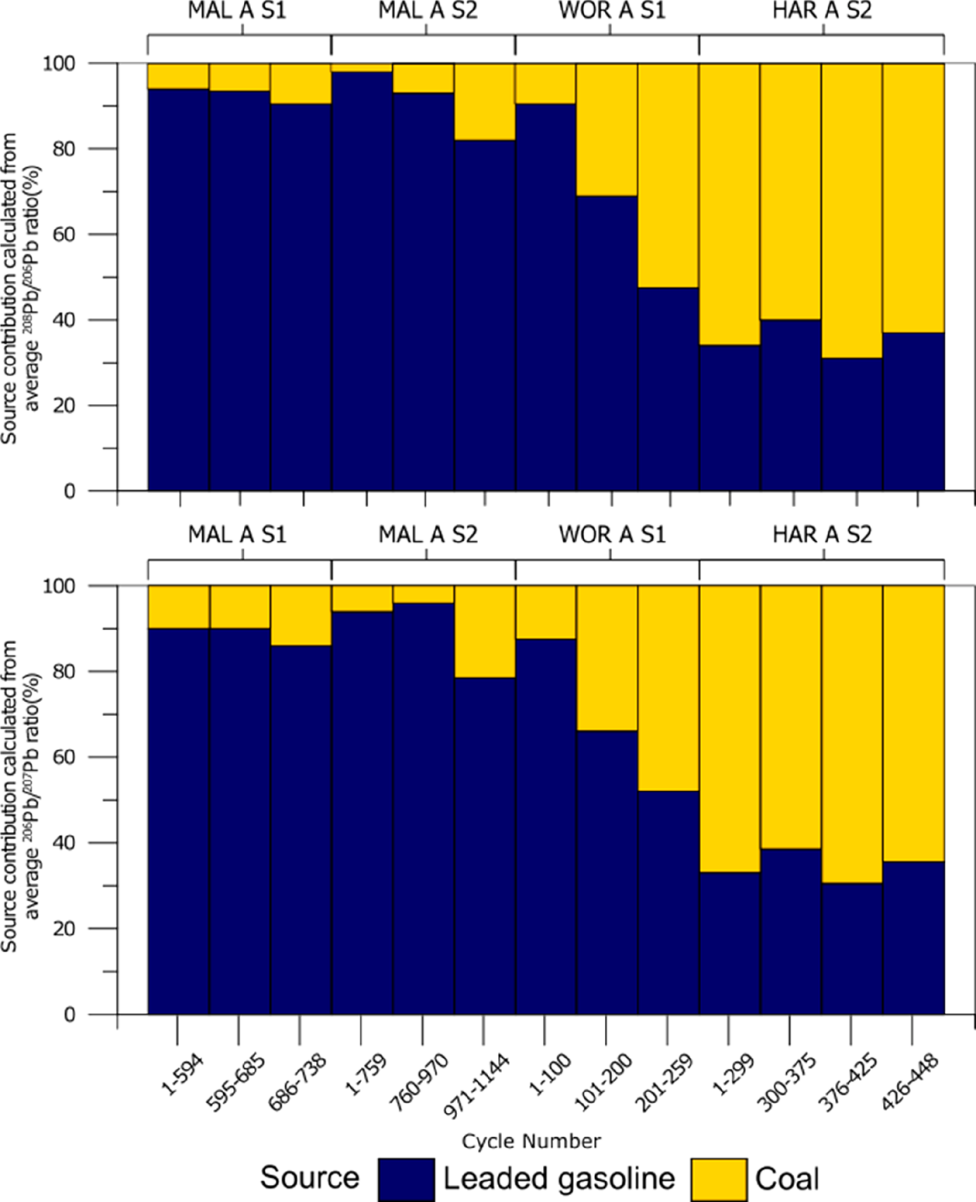
Mixing model-based contributions of two primary Pb sources to the black crusts studied here. For each head, the data cycles are divided into sections of similar Pb isotope compositions, with the average values modeled. Bar charts show modeled contributions from a British leaded gasoline (yellow) and a British coal (blue source for each of these sections). In the upper panel models were constructed using the ratio of ^206^Pb^/207^Pb, and from ^208^Pb/^206^Pb in the lower panel. Cf Table 3.

Overall, the isotopic data presented here can be explained by inputs from two primary sources of Pb, with the older black crust layers dominated by the signature of coal burning (e.g., the older portions of the first generation crust) and with outer layers containing Pb from leaded gasoline (e.g., the outer layers of some of the first generation crusts and the second generation crust). Assuming a two-component mixture and using measured isotope ratios of the respective sources (Table 3^49,65^), relative source contributions can be calculated for each of the LA-MC-ICP-MS cycle groups (Figure 4). This modeling indicates that first generation crusts (MAL and WOR) typically contain over 90% Pb from coal burning, with this shifting in crusts from second generation heads, where contributions from a set of modern pollution including but not limited to leaded gasoline (introduced after 1920) reach up to 60% (Figure 4).

**Table 3:** Results of the Grouping of Data Cycles for Each of the Heads Studied Here^a^

Head IDand cycle group	Average (±1SD) Pb (ppm)	Average ( ± 1SD) ^206^Pb/^207^Pb	Average (±1SD) ^208^Pb/^206^Pb	% Petrol from ^206^Pb/^207^Pb	% Coal from ^206^Pb/^207^Pb	% Petrol from ^208^Pb/^206^Pb	% Coal from ^208^Pb/^206^Pb
MAL A S1							
1–594	133.5 ± 81.1	1.177 ± 0.001	2.087 ± 0.001	6.0%	94.0%	10.0%	90.0%
595–685	534.6 ± 141.4	1.177 ± 0.001	2.088 ± 0.001	6.5%	93.5%	10.0%	90.0%
686–738	227.6 ± 62.6	1.173 ± 0.002	2.092 ± 0.003	9.5%	90.5%	14.0%	86.0%
MAL A S2							
1–759	54.9 ± 44.4	1.182 ± 0.004	2.084 ± 0.003	2.0%	98.0%	6.0%	94.0%
760–970	234.8 ± 151.8	1.175 ± 0.002	2.093 ± 0.004	7.0%	93.0%	14.0%	86.0%
971–1144	81.1 ± 49	1.163 ± 0.003	2.102 ± 0.004	18.0%	82.0%	21.5%	78.5%
WOR A S1							
1–100	31.2 ± 7.9	1.172 ± 0.002	2.089 ± 0.002	9.5%	90.5%	12.5%	87.5%
101–200	126.8 ± 85.9	1.147 ± 0.016	2.115 ± 0.015	30.5%	69.5%	34.0%	66.0%
201–259	41.8 ± 18.4	1.129 ± 0.004	2.132 ± 0.004	52.5%	47.5%	48.0%	52.0%
HAR A S2							
1–299	659.2 ± 313.9	1.107 ± 0.002	2.155 ± 0.002	66.0%	34.0%	67.0%	33.0%
300–375	654.6 ± 84.3	1.113 ± 0.001	2.148 ± 0.002	60.0%	40.0%	61.5%	38.5%
376–425	404 ± 235.9	1.103 ± 0.004	2.158 ± 0.004	69.0%	31.0%	69.5%	30.5%
426–448	42.4 ± 39.8	1.110 ± 0.004	2.152 ± 0.006	63.0%	37.0%	64.5%	35.5%

Source compositions	Average ^206^Pb/^207^Pb	Average ^208^Pb/^206^Pb	Citation
UK Leaded gasoline	1.067	2.0501	Monna et al., 1997^65^
UK Coal	1.18412	2.07576	Farmer et al., 1999^49^

a Also presented are the modelled source contributions for each group of data cycles, and the end member compositions used to calculate the source contributions.

Additionally, in first generation crusts, shifts from coal to gasoline are observed as the analyses move away from the host rock, suggesting the outermost (youngest) crust layers contain lower coal and higher gasoline Pb, consistent with a transition in the style of pollution the heads have been exposed to. Such a finding highlights how black crusts may indeed contain a chronology of pollution which is only resolvable with the ultrahigh-resolution nature of LA-ICP-MS analysis.

### Toward Establishing Black Crusts As Useful Long-Term Environmental Archive for Urban Air Pollution

Our study aims to establish black crusts as reliable long-term environmental archives for urban air pollution. To achieve this, we investigated the weathering history of a unique set of stone sculptures, analyzed known pollution trends, and conducted high-resolution Pb isotope analysis using LA-MC-ICP-MS for the first time. It is important to distinguish advanced high-resolution analysis of crustal stratigraphy, as illustrated in this paper, using LA-MC-ICP-MS, from bulk analysis. Stratigraphic analysis can reveal local changes in air pollution over time, while bulk analysis can contribute to the regional air pollution reconstruction. In this section, we integrate our study findings and observations into a broader discussion on black crusts as geochemical archives.

#### Factors Influencing the Formation and Suitability of Crusts As Geochemical Archives

Our study emphasizes the importance of considering specific factors in identifying “ideal” black crusts suitable for geochemical archive research. These factors encompass the (1) external environment, (2) interfaces between the atmosphere and crust surface as well as the crust-host material, and (3) the internal characteristics of the building material. By recognizing the interactions between these factors, we have identified the constituents of an “ideal” crust for geochemical archive purposes.

In this context, it is essential to consider the variations in the process drivers. At the interface between the atmosphere and the crust surface, these drivers are primarily influenced by environmental factors and the physicochemical properties of the crust surface. Conversely, processes occurring within the crust and at the intersection to the host material are further influenced by factors such as water-retaining porosity and chemistry in the bulk structure, as well as the presence of precipitated gypsum and carbonate phases. Understanding these distinctions contributes to a comprehensive assessment of black crust formation and its suitability as a geochemical archive.

#### External Environment

Regarding the external environment, we found that the exact timing of crust growth initiation and minimal disturbances from conservation interventions are critical for reliable archives. Ausset et al. (1998)^74^ demonstrated such examples for preindustrial air pollution stored in crusts from Arles (St Trophime) and Bologna (Palazzo d’Accursio) where crust growth initiation timing was estimated via previously exposed surfaces covered by later modifications of the architecture and a protective wax layer treatment, respectively. Del Monte et al. 2001^75^ exploited another opportunistic “stopped pollution clock” with the heads of the Kings of Juda from Notre Dame in Paris, which had been removed at a known point in time. In a similar manner, our work utilizes information on the timing of installation and displacement of three generations of sculptures in central Oxford. As the recognition of certain “ideal” crusts as reliable long-term outdoor archives grows, it is expected that other similar situations will also be recognized and utilized accordingly.

#### Environment, Crust, and Host Material Interface

The features of a black crust vary depending on the nature of their substrate.^24^ Black crusts are most common and best developed on calcareous substrates, where their boundary with the host substrate is often gradual (limestone, marble, and lime mortar). Nevertheless, black crusts are also recorded on other Ca-rich substrates like granite and trachyte, on which their boundary is typically sharp, and on silicate rocks near a leaching calcium-source.^76^ Although calcareous substrates are preferentially sampled because of their highest susceptibility to sulfation, the texture of the gypsum crust should be carefully analyzed. Gypsum crusts typically occur on the stone surface and in cracks. Crystals grow in cavities between or on top of mineral grains, sometimes resembling pseudomorphism in crystalline textures or components.^77^ Crystallization might be enhanced through the catalytic effects of airborne particles like soot or mineral grains like glauconite.^78−80^ Typically, two to three layers are distinguished on calcareous substrates, from outside to inside: (1) an opaque layer containing gypsum crystals and airborne particles, (2) a transparent or white layer, with gypsum crystals as (partial) alteration of the host substrate, (3) a zone with surface-parallel cracks, sometimes filled with gypsum that gradually evolves in the sound stone substrate.^48,80−85^ The original stone surface is typically located at the boundary of layer 1 and layer 2, which is emphasized by the distinct color change (from black to translucent) and by cathodoluminescence.^84^ This boundary marks the difference between the outer part of the crusts where accumulation of airborne particles in combination with precipitation is assumed and the inner part where dissolution–precipitation reactions are taking place. For stratigraphic analysis, it is important to focus on the outer (black) layer of deposition, as made evident by the results in this paper. Here, we emphasize the importance of analyzing the microscopic texture of the crust, distinguishing between deposition and alteration layers, and documenting the presence of artificial layers such as lead paint.^13^

In our study, we examined both laminar and framboidal crusts to encompass a broader range of morphological variations. The literature commonly distinguishes between these two crust morphologies, which are primarily controlled by the exposure regime rather than the substrate.^86^ Laminar crusts are typically found in sheltered areas, regions with occasional rain ingress, and areas with limited exposure to rain.^87,81^ They are characterized as thin and adherent, maintaining the substrate’s surface morphology without significant alterations. The stratification of laminar crusts suggests a simple deposition pattern with the oldest layers near the host rock and the youngest layers closer to the crust’s surface. These crusts are particularly suited for stratigraphic analysis and can also be used for bulk analysis.

On the other hand, framboidal crusts, also known as globular, dendritic, or cauliflower crusts, are predominantly observed in fully sheltered areas.^84^ They exhibit rosette-like gypsum crystals that enhance the entrapment of larger amounts of air pollutants. However, framboidal crusts are generally described as thick and prone to detachment from the surface. Siegesmund et al. (2007^88^) noted common breakdown features such as blistering and scaling, which can disrupt the stratification of accumulated pollutants. Consequently, the presence of framboidal crusts introduces complexities that may confuse the stratigraphic analysis, and there is a higher likelihood of older crusts having detached earlier, leading to increased uncertainty regarding the starting point of crust growth.

Average growth rates (or rather rates of change) of black crusts can range between 2–60 μm/annum.^89,90^ Given the inherent variability, our suggestion is to not focus on the thickness of the stratigraphy but on the stratigraphical geochemical fingerprints, as our results show that pollutants will accumulate in distinct layers. Furthermore, the quantification of layer thickness might vary, especially when the intersection of host stone and crust is not clearly defined.^89^

#### Mobility of Lead (Pb) in Black Crusts

The interface between the crust and the host substrate emerges as a pivotal factor in our study, particularly when establishing a reliable chronological record of air pollution based on crust stratigraphy. To achieve this, it is crucial to consider the mobility of elements used for dating such as Pb. The mobility of Pb and the factors that shape its behavior across diverse environmental contexts have been extensively studied. During the early stages of crust formation, including deposition as dust on urban surfaces or in urban sediments, Pb has been observed to exhibit greater mobility.^91−93^ This mobility is associated with various phases, including the water-soluble phase, the exchangeable/carbonate phase, and others.^91−93^ The mobility of Pb is further influenced by a range of factors, including the medium in which it is present. For instance, low pH values can enhance the solubility of Pb compounds from mineral surfaces.^91^ Surrounding environmental conditions, such as increased moisture, can also impact Pb mobility.^92^ Moreover, chemical processes like complexation and compound formation contribute to the overall dynamics of Pb mobility.^93^ For example, Astilleros et al. (2010^94^) describe a mechanism by which gypsum can effectively remove Pb from a solution. Through the coupled dissolution of gypsum and the subsequent precipitation of anglesite (PbSO_4_), a sparingly soluble salt, Pb can be immobilized within the structure of anglesite, reducing its mobility and potential environmental impact.^94^ Consequently, Pb can exhibit both mobile and immobile behavior under different circumstances.

#### Crust–Host Substrate–Interface

Another indicator of the mobility of Pb is its presence in the crust host substrate. While some studies demonstrate a clear distinction in Pb levels between the crust and the substrate (refs (10, 84, 95), and this study), others have detected significant amounts of Pb in the substrate,^23,26,96^ increased presence in subsurface cracks,^95^ or at the crust-host substrate interface.^97^ The geochemical affinity of Pb to carbonates under specific environmental conditions has been proposed to explain these observations.^91,93,97,98^ However, only one study^22^ reports higher Pb levels in the altered substrate compared to the crust, which aligns with the prevailing situation of decreasing Pb concentrations in the host rock within the first 100–300 μm.^95^

Various other factors may account for these observations beyond geochemical affinity, including (i) the presence of pre-existing crust acting as a barrier to Pb penetration into the substrate in urban surfaces that developed a crust before Pb pollution; (ii) favorable conditions such as high relative humidity or proximity to water (e.g., the Corner Palace in Venice (Italy) as discussed in Belfiore et al. (2013^26^); (iii) effects of gravity and horizontal surfaces;^23,99^ and (iv) specific weathering patterns where fissures and cracks allow gypsum to grow into the substrate, creating a sulfur-rich microenvironment.^22,95,97^ Additionally, redox potential, influenced by microbial activity and mineral precipitation processes, can also impact Pb mobility.^100−103^ Under reducing (redox) conditions, lead sulfides may form, which are relatively insoluble and immobile.

Drawing insights from our study in Oxford, we observed a strong and well-defined interface between high and low Pb concentrations that aligned with the interface between the crust and the host substrate. Taken together, our findings, along with the literature discussion, suggest that the well-defined interface between high and low Pb concentrations at the crust-host substrate boundary indicates minimal Pb mobility and notable chemical stability within the crust layers and their components.^84^ Therefore, we recommended where possible to include the substrate close to the crust in the overall analysis to show the distribution of Pb and to account for its potential mobility.

### Environmental Implications

Our study contributes to a deeper understanding of localized pollution levels, specifically in densely populated urban environments. While global pollution levels are often assessed using methods, such as ice cores, our research focuses on capturing and analyzing pollution patterns at a more local scale. By highlighting the valuable role of black crusts as archives of geochemistry, we shed light on their significance in investigating historical air pollution, particularly in urban settings where alternative archives may be limited or unavailable.^114,113−115^ Our data shows that it is possible to produce a fine resolution pollution record from the geochemistry in the stratigraphy of the black crusts when specific built structure configurations are exploited for “calibration” to establish a reliable chronology. Accordingly, our summary table (ST2) has been carefully designed to offer valuable guidance, aiming to enhance the reliability of this methodology in this field.

Furthermore, by examining the behavior of Pb in black crusts, our study contributes to a deeper understanding of its dynamics within these crusts and increases their potential as reliable geochemical archives for studying urban air pollution. We have discussed multiple factors that underscore the intricate nature of Pb mobility and its interactions with the environment. It is important to recognize that Pb can display both mobile and immobile behavior, depending on the specific circumstances at play. Comprehending these dynamics is crucial, especially considering the enduring legacy of lead pollution. Notably, despite the global ban on leaded petrol in 2021 (UN environment program), the legacy of past and present air pollution continues to pose significant challenges for urban environments worldwide.^104−111^

By integrating black crusts as important indicators, our study contributes to a more holistic approach that complements multiple sources of evidence. This comprehensive approach enhances our understanding of historical urban pollution patterns,^112,116^ revealing the detailed dynamics and trends of air pollution.

This enhanced understanding can aid in assessing the differential, multidimensional effects of air pollution, inferring the long-term environmental effects of pollutants and supporting the implementation of effective strategies to mitigate impacts on human health, local ecosystems, and biodiversity. The pollution record can serve as a reference point for assessing the success of pollution reduction measures over time. This could provide valuable insights into the effectiveness of various environmental policies and regulations. Such an enriched understanding, underpinned by historical data, might not only shape policies related to air pollution control, urban planning, and environmental conservation but also guide environmental risk assessment and management strategies in the future.

## Supporting Information Available

Geographical locations of the stone head sculptures in this study (exact GIS are not provided for GPDR reasons; Figure S1). Crust sampling areas on stone head sculptures (Figure S2). Three isotope lead (Pb) plot comparing the signature of UK coal to ores smelted in the south of England during the industrial revolution (Figure S3). Analyses of standard materials NIST SRM612 and BCR-2G (Table S1). Overview of crucial factors to consider and report when investigating black crusts as long-term, geochemical archive for urban pollution with respective references for examples (Table S2). (PDF)
