# Supplementary material for: The Historic Built Environment As a Long-Term Geochemical Archive: Telling the Time on the Urban “Pollution Clock”
Source: Environ Sci Technol. 2023 Jul 12;57(33):12362–75. doi: 10.1021/acs.est.3c00153 (PMC10448721; doi:10.1021/acs.est.3c00153)
Supplement: Supplementary file 1 — es3c00153_si_001.pdf [file es3c00153_si_001.pdf]

SUPPORTING INFORMATION (SI)

The historic built environment as a long-term geochemical archive: Telling the time on the urban 'pollution clock'

Katrin Wilhelm[a], Jack Longman[b, c], Christopher D. Standish[d], Tim De Kock[e]*

[a] Oxford Resilient Buildings and Landscapes Laboratory (OxRBL), School of Geography and the Environment, University of Oxford, South Parks Road, Oxford, OX1 3QY, UK; Katrin.wilhelm@ouce.ox.ac.uk

[b] Marine Isotope Geochemistry, Institute for Chemistry and Biology of the Marine Environment (ICBM), University of Oldenburg, Carl-von-Ossietzky-Str. 9-11, 26129 Oldenburg, Germany; jack2.longman@northumbria.ac.uk

[c] Department of Geography and Environmental Sciences, Northumbria University, Newcastle-upon-Tyne, NE1 8ST, United Kingdom

[d] School of Ocean & Earth Sciences, University of Southampton, National Oceanography Centre, European Way, Southampton, SO14 3ZH, UK; C.D.Standish@soton.ac.uk

[e] Antwerp Cultural Heritage Sciences (ARCHES), Faculty of Design, University of Antwerp, Blindestraat 9, 2000 Antwerp, Belgium; Tim.DeKock@uantwerpen.be

NUMBER OF PAGES: S7

NUMBER OF FIGURES: 3

NUMBER OF TABLES: 2

Table S1. Analyses of standard materials NIST SRM612 and BCR-2G

Analysis ID	Pb (ppm)	2SE	$^{206}Pb/^{207}Pb$	2SE	$^{208}Pb/^{206}Pb$	2SE
NIST612-1	40.1	0.7	1.10263	0.00008	2.16462	0.00009
NIST 612-2	40.1	2.8	1.10260	0.00008	2.16467	0.00017
NIST 612-3	43.2	1.1	1.10249	0.00011	2.16447	0.00018
NIST 612-4	43.6	1.1	1.10270	0.00010	2.16415	0.00021
Average	41.8		1.10260		2.16448	
2SD	3.8		0.00017		0.00047	
2SD %	9.1		0.01550		0.02186	
BCR2G-1	7.2	0.2	1.20037	0.00034	2.06472	0.00034
BCR2G -2	13.4	0.8	1.20000	0.00016	2.06476	0.00029
BCR2G -3	13.4	0.4	1.19990	0.00016	2.06495	0.00021
BCR2G -4	13.1	0.3	1.19989	0.00016	2.06499	0.00023
Average	11.8		1.20004		2.06485	
2SD	6.1		0.00045		0.00027	
2SD %	52.2		0.03771		0.01311	

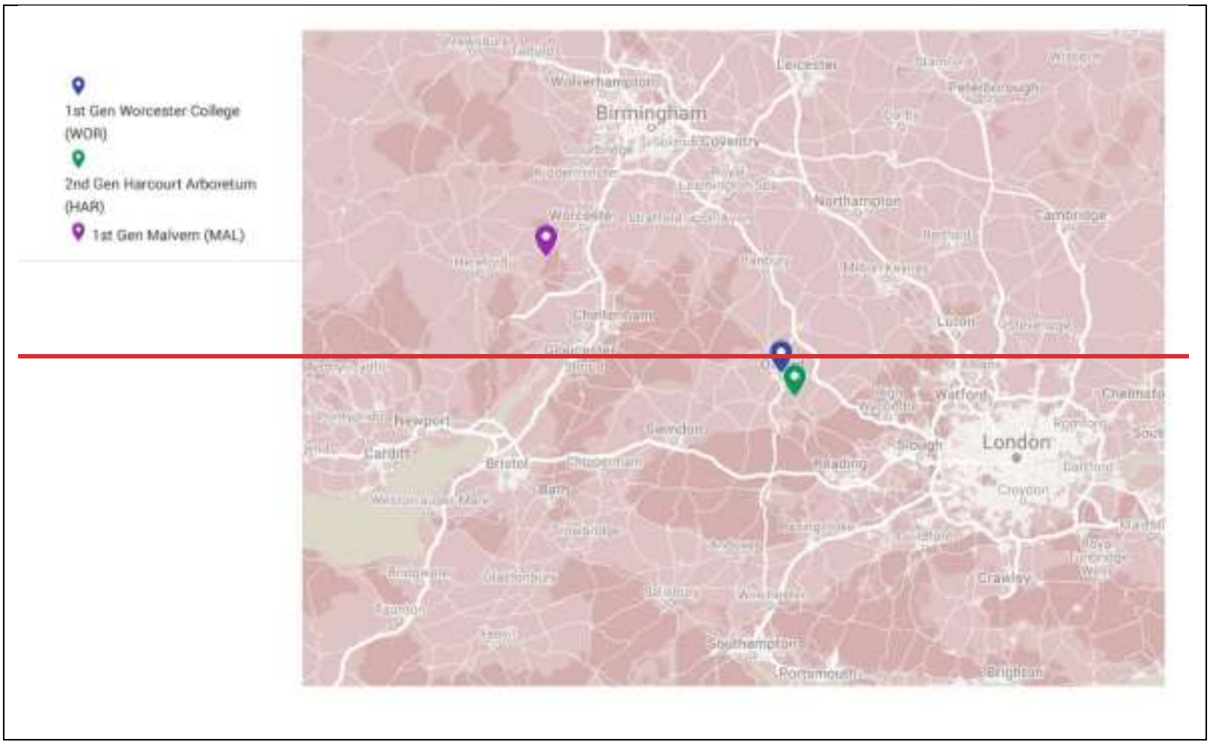

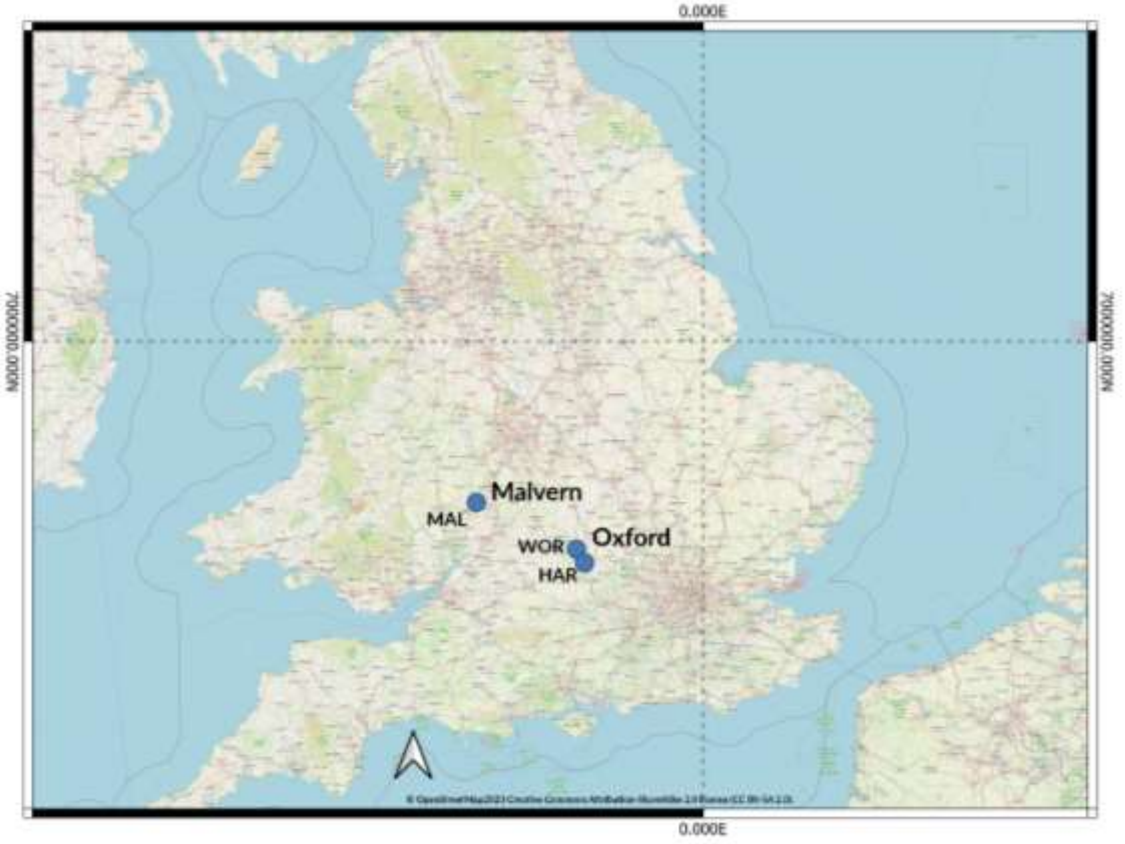

Figure S1. Geographical locations of the stone head sculptures in this study (exact GIS are not provided for GPDR reasons; basemap source: © OpenStreetMap2023 Creative Commons Attribution-ShareAlike 2.0 license (CC BY-SA 2.0).).

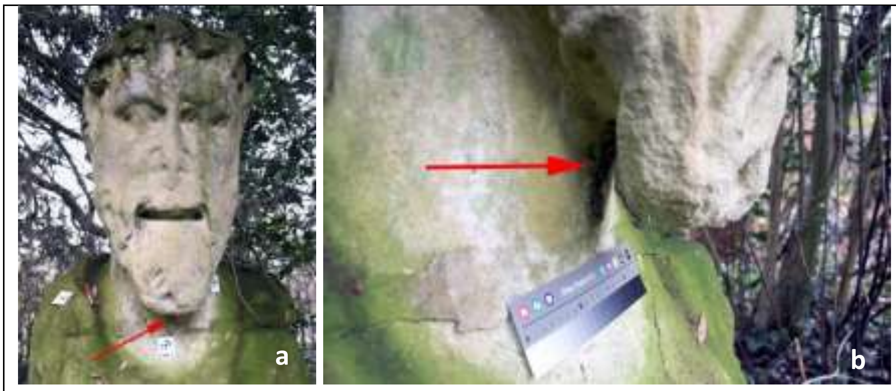

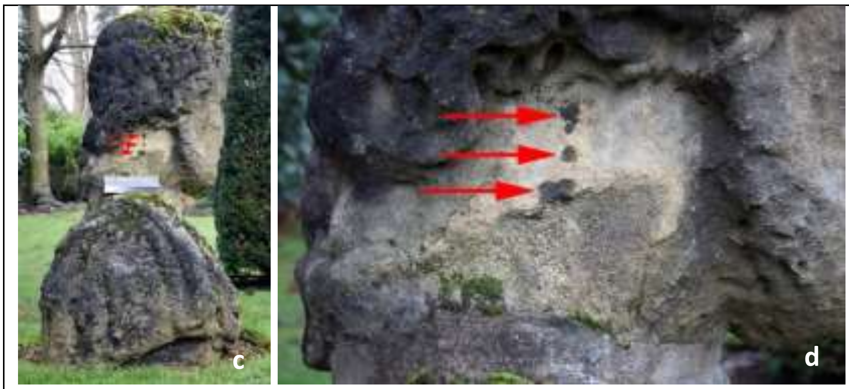

Figure S2. (a) 1st generation stone head sculpture A, Old Country House, Malvern (MAL S1-S2); (b) detail of crust sampling area for the same head MAL S1-S2 (c) 1st generation Head sculpture A, Worcester College, Oxford (WOR A S1), (d) detail of crust sampling area for the same head WOR A S1 (red arrows indicate sampling locations) (c) Wilhelm 2019,2020)..

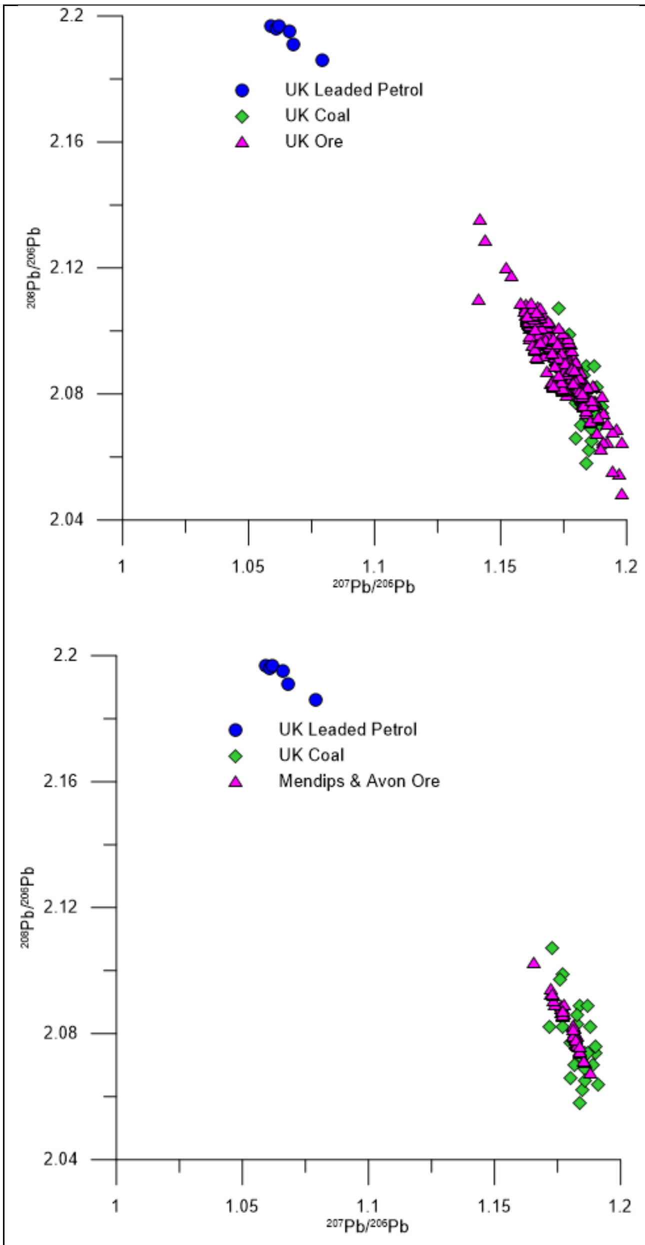

Figure S3. Three isotope lead (P~~p~~b) plot comparing the signature of UK coal to ores smelted in the south of England during the industrial revolution. Importantly, the ore and coal isotope signatures overlap, meaning we are unable to distinguish between the two sources in this work. ~~Isootpe~~Isotope data for leaded petrol are from Monna et al. (1997), with UK~~k~~ coal ~~compsiitons~~compositions from Farmer at el. (1999). Mendips and Avon ore compositions are form the OXALID database (https://oxalid.arch.ox.ac.uk/).

Table S2. Overview of crucial factors to consider and report when investigating black crusts as long-term, geochemical archive for urban pollution with respective references for examples

Factor (alphabetical)	*Theme*	*What to report and [format]?*	*Why important?*	*Reference(s)*
Aspect	Built env.	[N, E, W, S] and sub-directions	-previous research has shown that the accumulation of air pollutants on built structures surfaces varies according to aspect and crust formation occurs preferably on moderately exposed or sheltered areas	17, 22, 25, 29, 83, 91, 91
Climate	Nat. env.	Köppen climate classification	-allows for comparison of similar climate zones<br>-allows for future predictions in light of climate change	31, 115, 116
Conservation history of the built structure	Built env.	-All known dates of conservation interventions<br>-Major incidents such as war, natural hazards, fire, etc.<br>-effects of structural integrity	-allows for improved accuracy when analysing the crust stratigraphy	17, 22, 29, 91, 117, 117
Date of sampling	Analysis	[dd/mm/yyyy]	-to determine a chronology end datum point of the respective study	25, 118
Datum point for crust forming	Built env.	-records of surface cleaning<br>-replacement stones<br>-removal date of an artefact/sculpture into an archive<br>-date of architectural modification which obscures surfaces from the environment	- to determine the time period of crust formation and pollution accumulation	28, 74, 75
Environment classification	Nat-Built env.	Urban, marine urban, industrial urban, rural, size of city etc.	-it allows to categories pollution sources and weathering processes	17, 22, 25, 29, 83, 119
Geolocation	Nat-Built env.	GPS coordinates	-it allows for a precise mapping of the pollution record stored in the crust<br>-crust forming processes might be different for northern (colder) vs southern climate (warmer)	28, 120, 121, 121
Geometry and surface morphology of the host structure	Built env.	-Flat areas, exposed ledges, cornices, arcades, sculpture, surface finishes such as tooling, undercuts; sheltered and exposed, etc.<br>-qualitative and quantitative description<br>-Surface roughness measurements	-the geometry of the built structure has an influence on the microclimate and moisture regimes which in turn affect crust formation processes	83,122,123, 84<br>83,122,123, 84
Height	Built env.	[m]	-pollutant amount and composition might be different depending on height<br>-The lower base (up to 1.50 m) should be excluded from sampling as this is the most dynamic in terms of	24, 83, 124, 98, 61, 124, 98, 61

Factor (alphabetical)	Theme	What to report and [format]?	Why important?	Reference(s)
			weathering processes with salt contamination and various sources of water (spray, ground water, run-off<br>-hight variations up to 5m are considered negligible, only considerable heights above ground (e.g., 35m) might show statistical differences	
Heterogeneity at block scale	Substrate	Homogenous vs heterogeneous	-might indicate past disturbance<br>-might indicate different microclimatic levels<br>-level will inform the number of samples to represent the overall built structure	125, 125, 126, 126
Host rock lithology/mineralogy	Substrate	Ideally the stone description should follow common standard such as Folk 1959 and Dunham 1962; alternatively, any known information of mineralogy should be added (and go beyond basic descriptions such as limestone, granite, etc.)	-crust forms in different ways on granite, sandstone, marble, travertine and limestone with sedimentary stone and limestone more prone to crust formation<br>-for the same rock type the mineralogical differences might be less influential to the crust mineralogy and crust morphology might play a more important role to trap pollution	29, 83, 127, 128, 83, 127, 128
Level and type of pollution	Built env.	-environmental air quality records<br>-history of pedestrianisation<br>-traffic load<br>-adjacent industry<br>-major incidents such as fires, flooding, storms<br>-trace metals, PAHs, particulate matter	-while usually the level of pollution is reflected in the pollutant composition of the crust, it is not known how much exactly of the amount of pollution in the air at a given point in time is accumulating in the crust. However, the exposure to high amounts of pollutants through vehicular traffic and/or industry results in higher reactivity and thicker layers have been reported respectively.	20, 83, 129, 83, 129
Morphology of crust	Crust	No unified terminology exists, and the surface morphology of crusts has been described as smooth, laminar, globular, dendritic, cauliflower and framboidal or rock coatings	-the different crust morphologies interact with air pollution in different ways, e.g., framboidal crusts exhibit rosette-like gypsum crystals which help entrap larger amounts of air pollutants	82, 83, 84, 130, 131, 84, 130, 131
Orientation	Built env.	Horizontal, vertical, tilted, concave, convex	-crusts formation occurs on vertical, tilted, and horizontal surfaces<br>-the accumulation of pollutants on horizontal surface is higher because of lower impact of wash-/run-off	25, 22, 132, 132
Period of exposure	Built env.	-archival records<br>-institutional knowledge<br>-traces of original surfaces such as toolmarks, dates on gravestones, mason's marks, etc.	-the more is known about the history of the building and respective interventions the higher the accuracy of the reconstruction of a pollution chronology using black crusts	17, 22, 29, 83, 91, 98, 117, 83, 91, 98, 117

Factor (alphabetical)	*Theme*	*What to report and [format]?*	*Why important?*	*Reference(s)*
Porosity of the host substrate	Substrate	-direct through sampling and laboratory analysis (e.g., thin sections, water uptake, etc.)<br>-indirect through in-situ measurements (water uptake, air-permeability, etc.)	-a high porosity host rock and gypsum crust offers a larger surface area for enhanced chemical interaction with the environment which (might) result in thicker crusts	22, 23, 119,
Porosity of the crust	Crust	-direct through sampling and laboratory analysis (e.g., thin sections, water uptake, etc.)<br>-indirect through in-situ measurements (water uptake, air-permeability, etc.)	-a high porosity of the gypsum crust offers a larger surface area for enhanced chemical interaction with the environment which (might) result in thicker crusts (typically cauliflower crust morphologies)<br>-a low porosity might temporarily function as a protective layer	105, 84, 133 105, 84, 133
Scale/range	Nat-Built env.	-micro, meso, macro<br>-local, regional, national, international, global	-air pollution sources contribute at the local, regional, national and international range.<br>- the forming of gypsum crust occurs both in urban and rural areas in all climatic conditions and has been linked to atmospheric background concentrations of SO2<br>-there is an indication that local sources in close proximity such as traffic contribute stronger to the pollutant composition and particle deposition compared to pollution sources which might be situated further away from the reactive urban surface such as industry	20, 129
Secondary weathering processes (cause)	Crust / Substrate	run off, etc.	-secondary processes need to be considered to increase the accuracy of the pollution chronology derived from the crust stratigraphy	117, 84, 117, 134, 84, 134
Secondary alteration (effect)	Crust / Substrate	redistribution of limewash, render and mortar and/or crust material and pollutants through run off, wash-out, erosion, etc.	-wash off from metal parts might contribute to the trace metal analysis	84, 84, 134, 134
Water sources	Nat-Built env.	Rain, sea spray, groundwater, fog, plant watering, broken gutters, relative humidity (RH%)	-the supply and type of water will determine crust-substrate system dynamics	10, 135
